# The possibility of using shogaol for treatment of ulcerative colitis

**DOI:** 10.22038/IJBMS.2018.28616.6932

**Published:** 2018-09

**Authors:** Snur MA Hassan, Ali Hussein Hassan

**Affiliations:** 1 Department of Anatomy and Pathology, College of Veterinary Medicine, Sulaimani University, Kurdistan, Iraq

**Keywords:** Albino mice, Colitis, Dextran sodium sulfate, IBD, Shogaol, 6-thioguanine

## Abstract

**Objective(s):**

This study aimed to investigate the effect of Shogaol on dextran sodium sulfate (DSS)- induced ulcerative colitis (UC) in mice compared to an immune-suppressant chemotherapeutic medicine, known as 6-thioguanine (6-TG).

**Materials and Methods:**

Thirty-six adult BALB/c mice were divided into six groups: group 1 (positive control): no DSS exposure and no treatment; group 2 (negative control): DSS exposure without treatment; group 3 (vehicle control): DSS exposure and olive oil treatment; group 4: DSS exposure and 0.3 mg/kg 6-TG treatment; group 5: DSS exposure and 20 mg/kg Shogaol treatment; and group 6: DSS exposure and 40 mg/kg Shogaol treatment. At day 16, the mice were euthanized and UC was evaluated according to colon length, histologically index score and expression scores of the epidermal growth factor receptor (EGFR).

**Results:**

The disease activity index (DAI) and histological index scores of mice treated with 40 mg/kg body weight (BW) Shogaol were approximately lower than the corresponding scores of mice treated with 6-TG. In addition, the rate of healing in the former mice was approximately 3 folds higher than that of the latter ones as indicated by the lack of EGFR expression in colonic glands and macrophages.

**Conclusion:**

These findings showed that the therapeutic effect of 40 mg/kg BW Shogaol could be better than 6-TG in the treatment of UC, and it may draw the attention regarding the priority of using this cheap plant-derived substance for treatment of the inflammatory bowel diseases because treatment with 6-TG is usually associated with adverse side effects.

## Introduction

The risk of disease in the gastrointestinal tract is high because of the continuous exposure to numerous bacteria as well as food-derived and environmental toxins (1). Crohn’s disease and ulcerative colitis (UC), two major types of inflammatory bowel diseases (IBD) with multifactorial etiology, are characterized by both acute and chronic inflammation of the intestine and cause an enormous burden to public health (2). In the last few decades, various models of experimental IBD have been developed to characterize the complexity of IBD pathogenesis, delineating underlying molecular mechanisms and to improve treatment options (3, 4).

Dextran sodium sulfate (DSS), a water-soluble, negatively charged, sulfated polysaccharide with a highly variable molecular weight ranging from 5 to 1400 kDa, is employed to induce colitis in mouse, the most widely used animal model of colitis (5). DSS-induced colitis in mice is a suitable model characterized by morphologically and histologically features similar to acute and chronic UC in humans such as diarrhea, hematochezia, weight loss, mucosal ulceration, and extensive mucosal damage (6, 7).

Patients with IBD are conventionally treated with steroidal and non-steroidal anti-inflammatory drugs, immune-suppressants, and/or antibiotics; however, these medications temporarily induce and maintain remission in only 45% of patients. In addition, they have numerous side effects, and drug tolerance has been observed in some patients (8, 9); therefore, the exploration of new medications for IBD patients is to be maintained.

Shogaol, one of the phenolic constituents of ginger, has an antimicrobial, antioxidant, anti-inflammatory, analgesic, antipyretic, anti-diabetic, antiemetic, antitussive, and hypotensive effects (10) and recently, there has been a growing interest in Shogaol for its potential effects against cancers, such as ovarian, lung, skin, colon and liver cancers (11).

Epidermal growth factor (EGF) that is the prototypical ligand for EGF receptor (EGFR) is secreted by the submandibular and Brunner’s glands under physiological conditions (12). However, it can be produced by other cell types under pathological conditions, such as the intestinal epithelial cells in response to injury (13). Biological functions of EGFR include promotion of cellular proliferation, differentiation, migration, and survival (14).

The present study aimed to investigate the possible protective effect of Shogaol on DSS-induced colitis in BALB/c mice in comparison with 6-thioguanine (6-TG), an immunosuppressant chemotherapy, which is conventionally used for UC, based on scoring of disease activity index (DAI), histological index and EGFR expression.

## Materials and Methods

***Animals and treatments***

Thirty-six adult, male and female BALB/c mice weighing 25-30 g were purchased from the Animal House at the College of Veterinary Medicine, University of Sulaimani (Sulaimaniyah Governorate, Iraq), accommodated at the same house in temperature and light-controlled animal facilities and permitted consumption of tap water and standard food *ad libitum*. All mice-involving procedures in this study were carried out humanely and were performed in accordance with the principles outlined in the Guide for the Care and Use of Laboratory Animals and with the approval of the Ethics Committee at the College of Veterinary Medicine, University of Sulaimani.

All mice, except the negative control (group 1), were exposed to 5% DSS (molecular weight 40 kDa; Carl Roth GmbH+ Co. KG) via drinking water (5% weight/volume) for 5 days to induce UC (15). The treatment-containing water was changed every day. Following that, the DSS-exposed mice were divided into 5 groups (group 2 to 6) as follows: group 2 (positive control) that was left without treatment, group 3 (vehicle control group) treated with 1 ml/kg body weight (BW) olive oil (8873.1-Carlroth), group 4 treated with 0.3 mg/kg BW 6-TG (16) prepared in a vial of sterile water (Biochem, Chemopharma, France), groups 5, and 6 treated respectively with 20 mg and 40 mg/kg BW Shogaol (≥90%-Sigma-Aldrich). The Shogaol was dissolved in olive oil as a vehicle.

All treatments (other than the 5% DSS) were given as a single daily dose by oral gavages (a total of 8 doses for each treatment during 10 days; i.e., four days treatment with one-day interval). 

**Table1:** Disease activity index score (DAI score).

**Score**	**Weight loss**	**Stool consistency**	**Rectal bleeding**
**0**	None	Normal	no bleeding
**1**	1-5%	-	-
**2**	5-10%	loose stool	Mild bleeding
**3**	10-15%	-	-
**4**	more than 15%	watery diarrhea	Prominent bleeding

Sum of scores: a range of 0-12.

**Table 2:** Histological scoring of ulcerative colitis

**Inflammatory cell infiltrate (Score 1)**			**Intestinal architecture (Score 2)**		
**Severity**	Extent	Score value	Epithelial changes	Mucosal architecture	Score value
**Normal**	-	0	Intact	-	0
**Mild**	Mucosa	1	Focal erosions	-	1
**Moderate**	Mucosa and submucosa	2	Ulcerations	Focal ulcerations	2
				Extended ulcerations ± granulation tissue ± pseudopolyps	3
**Marked**	Transmural	3			

Sum of scores 1 and 2: a range of 0-6.

**Table 3:** Changes in body weight of mice in all groups during the experiment

**Group**	**Control negative**	**Control positive** **(DSS exposure)**	**Vehicle control**	**DSS exposure and 6-TG treatment**	**DSS exposure and 20 mg/kg BW Shogaol treatment**	**DSS exposure and 40 mg/kg BW Shogaol treatment**
**Day 1** **starting weight**	24.50 ^a^ ±0.16	27.83 ^a^ ±0.47	21.00 ^a^ ±0.28	26.83 ^a^ ±0.47	24.50 ^a^ ±0.22	28.33 ^a^ ±0.21
**Day 5** **post-DSS exposure weight**	24.83 ^a^ ±0.22	22.50 ^b^ ±0.71	18.50 ^b^ ±0.42	23.50 ^b^ ±0.22	21.83 ^b^ ±0.47	25.66 ^b^ ±0.49
**Day 16** **post-treatment weight**	25.33 ^a^ ±0.21	21.83^ b^ ±0.60	18.66 ^b^ ±0.21	24.00 ^a^ ±0.36	23.00 ^a^ ±0.36	27.66 ^a^ ±049

The body weights are expressed by mean ± standard error.

Means of body weight that do not have similar alphabetical letters vary from each other (*P < 0.05*). DSS: Dextran sodium sulfate, BW: Body weight, 6-TG: 6-thioguanine.

**Table 4:** Average colon lengths in mice of different groups at the end of the study (mean±SD)

**Group**	**Average colon length ±SE (cm)**
**Control negative**	12.33 ±0.24 ^a^
**Control positive (DSS exposure)**	7.22 ±0.10 ^b^
**DSS exposure and olive oil treatment (vehicle control)**	8.21 ±0.08 ^b^
**DSS exposure and 6-TG treatment**	9.18 ±0.18 ^a^
**DSS exposure and 20 mg/kg BW Shogaol treatment**	9.96 ±0.19 ^a^
**DSS exposure and 40 mg/kg BW Shogaol treatment**	11.65 ±0.10 ^a^

Average colon lengths that do not have similar alphabetical letters vary from each other (*P < *0.05). DSS: Dextran sodium sulfate, BW: Body weight, 6-TG: 6-thioguanine.

**Figure 1:**
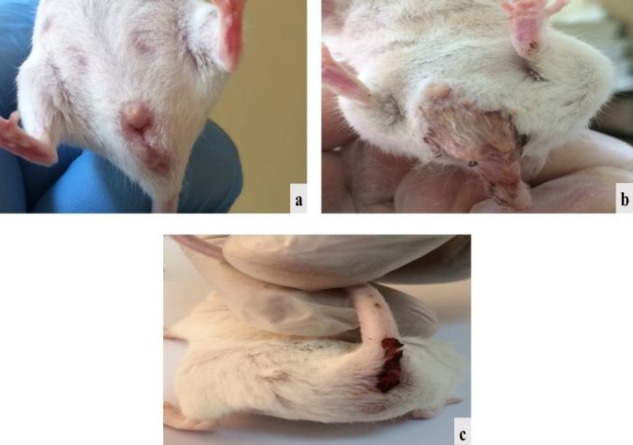
Mice with no rectal bleeding (a and b) belonging respectively to group 1 (negative control) and group 5 (DSS exposure + 40 mg/kg BW Shogaol treatment) compared to a distinctive rectal bleeding (c) in a mouse belonging to group 2 (positive control). DSS: Dextran sodium sulfate, BW: Body weight

**Figure 2:**
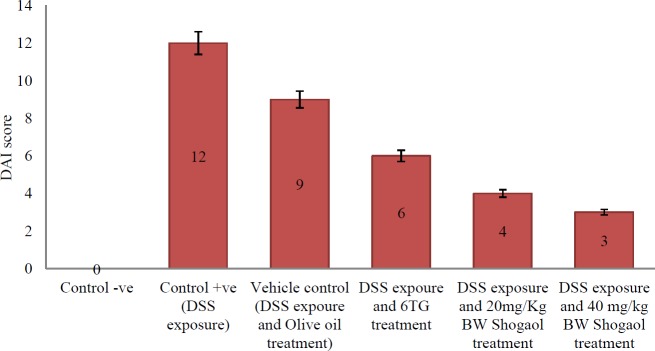
Disease activity index (DAI) scores in all groups of mice as an indicative for ulcerative colitis. DSS: Dextran sodium sulfate, BW: Body weight, 6-TG: 6-thioguanine

**Figure 3:**
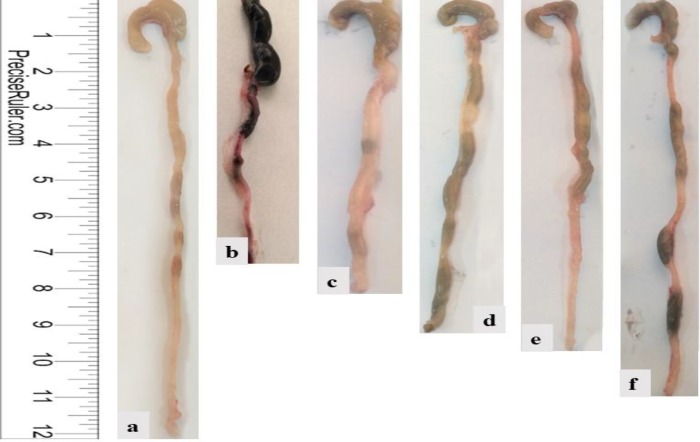
Colon length as an indicator of ulcerative colitis. a: Control –ve, b: Control +ve (DSS exposure), c: Vehicle control group (DSS exposure and olive oil treatment), d: DSS exposure and 6-TG treatment, e: DSS exposure and 20 mg/kg BW Shogaol treatment, f: DSS exposure and 40 mg/kg BW Shogaol treatment. DSS: Dextran sodium sulfate, BW: Body weight, 6-TG: 6-thioguanine

**Figure 4:**
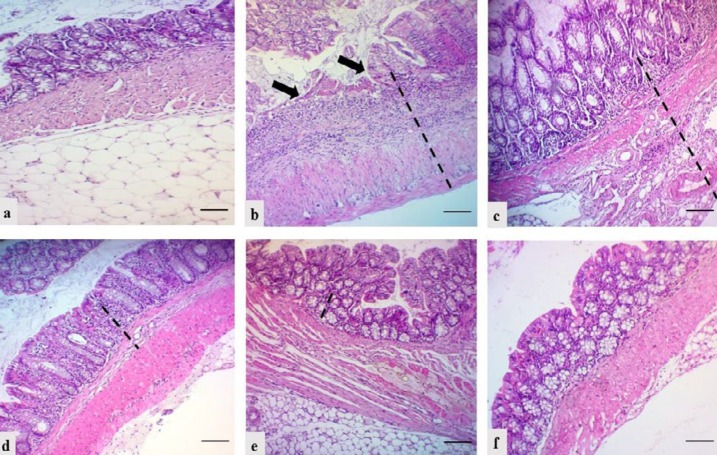
Microscopic view and the total histological index score of the proximal colon of mice in all groups of the current study.a: Group 1 (negative control): Intact epithelium with normal epithelial cells infiltration (Sum score 0); b: Group 2 (control +ve “DSS exposure without treatment”): Focal epithelial ulceration (black arrows) with transmural infilteration of inflammatory cells (Sum score 5); c: Group 3 (vehicle control group): Intact epithelial surface with transmural infiltration of inflammatory cells (Sum score 4); d: Group 4 (DSS exposure and 6-TG treatment): Intact epithelial surface with moderate infilteration of inflammatory cells in mucosa and submucosa (Sum score 2); e: Group 5 (DSS exposure and 20 mg/kg BW Shogaol treatment): Intact epithelium with mild infilteration of inflamatory cells in mucosa only (Sum score 1); f: Group 6 (DSS exposure and 40 mg/kg BW Shogaol treatment): Intact epithelium with no inflammatory cells infilteration (Sum score 0). H&E stain; Black dash line indicated the extent of inflammatory cells infiltration; scale bar 100 μm. DSS: Dextran sodium sulfate, BW: Body weight, 6-TG: 6-thioguanine

**Figure 5:**
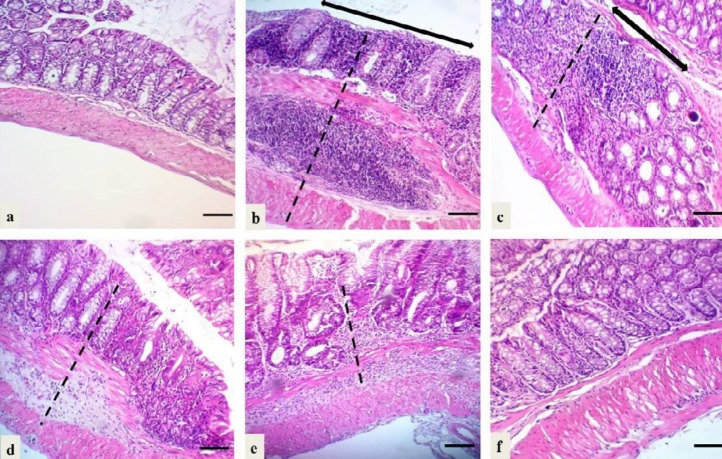
Microscopic view and the total histological index score of the distal colon of mice in all groups of the current study. a: Group 1 (negative control): Intact epithelium with normal epithelial cells infiltration (Sum score 0); b: Group 2 (control +ve “DSS exposure without treatment”): Extensive epithelial ulceration (black arrows) with transmural infilteration of inflammatory cells (Sum score 6); c: Group 3 (vehicle control group): Focal epithelial erosion (black arrow) with transmural infiltration of inflammatory cells (Sum score 4); d: Group 4 (DSS exposure and 6-TG treatment): Intact epithelial surface with moderate infilteration of inflammatory cells in mucosa and submucosa (Sum score 2); e: Group 5 (DSS exposure and 20 mg/kg BW Shogaol treatment): Intact epithelium with moderate infiltration of inflammatory cells in mucosa and submucosa (Sum score 2); f: Group 6 (DSS exposure and 40 mg/kg BW Shogaol treatment): Intact epithelium with mild infiltration of inflammatory cells in the mucosa (Sum score 1). H&E stain; Black dash line indicated the extent of inflammatory cells infiltration; scale bar 100 μm. DSS: Dextran sodium sulfate, BW: Body weight, 6-TG: 6-thioguanine

**Figure 6:**
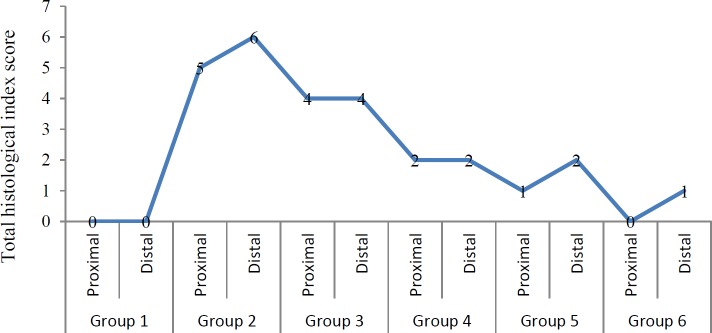
Total histological index score of proximal and distal colonic segments as an indication for ulcerative colitis in mice. Group 1: Negative control, Group 2: Positive control (DSS exposure without treatment), Group 3: Vehicle control group, Group 4: DSS exposure and 6-TG treatment, Group 5: DSS exposure and 20 mg/kg BW Shogaol treatment, Group 6: DSS exposure and 40 mg/kg BW Shogaol treatment. DSS: Dextran sodium sulfate, BW: Body weight, 6-TG: 6-thioguanine

**Figure 7:**
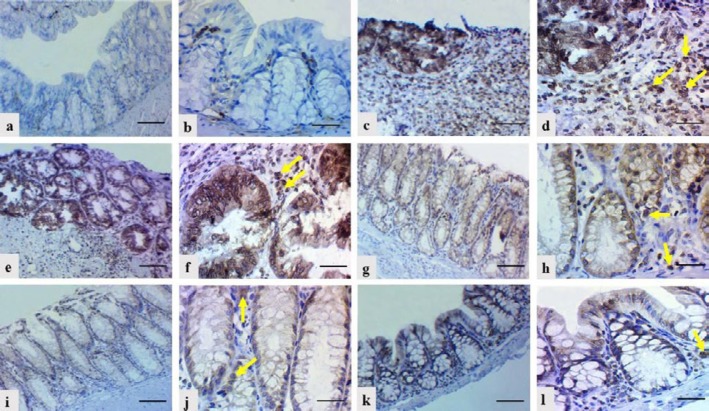
Epidermal growth factor receptor (EGFR) expression (yellow arrows) in macrophages and in epithelial lining cells of mucosal glands in colonic tissue sections of different mice groups. a and b: Negative expression (Sum score 0) in negative control group; c and d: Strong expression (Sum Score 9) in positive control group (DSS exposure without treatment); e and f: moderate expression (Sum Score 6) in vehicle control group; g and h: moderate expression (Sum Score 4) in group of DSS exposure and 6-TG treatment; i and j: weak expression (Sum Score 1) in group of DSS exposure and 20 mg/kg BW Shogaol treatment; k and l: negative expression (Sum Score 0) in group of DSS exposure and 40 mg/kg BW Shogaol treatment. Scale bar 100 μm. DSS: Dextran sodium sulfate, BW: Body weight, 6-TG: 6-thioguanine

***Assessment of colitis***

*Body weight measurements and disease activity index score*

The BW was measured 3 times over the 16 days period of the experimental duration (starting weight, post-DSS exposure weight, and post-treatment weight), and the mice were inspected for consistency of their stool and presence of rectal bleeding around the anus. In addition, DAI score (Table 1) was used to describe the severity of UC (17).

*Colon measurements and histological scoring*

At the end of the experimental period, the mice were anesthetized by ketamine and xylazine then euthanized by cervical dislocation. The abdomen was opened and the entire colon was resected, placed on a clean filter paper and its length (in a relaxed position without stretching) was measured by a ruler. Following that, the colon was emptied from its contents, dissected longitudinally and washed by neutral buffered saline. The proximal and distal colon portions were separated from each other, placed on separate filter papers for 2 min and immediately fixed in 10% neutral buffered formalin for 24 hr.

Subsequently, colon samples were obtained and undergone a series of histopathological preparations. Transverse colon tissue sections (4 μm thick) were obtained using a rotary microtome, stained with hematoxylin and eosin and examined by different magnifying powers of light microscopy (Leica, Germany). Histological index score (Table 2) was used to assess the severity of UC in mice of the different groups according to the histopathological morphology (18). 

*Immunohistochemistry staining*

Colon sections (4 μm thick) were fixed on the positively charged slide and allowed to dry for 1 hr at room temperature followed by 1 hr in an incubator at 60 ^°^C. The sections were deparaffinized and rehydrated with xylene and graded alcohol solutions. Antigen retrieval was performed by boiling in the pressure cooker for 20 min in citrate buffer. Endogenous peroxidase activity was blocked by dipping the slides in 0.3% hydrogen peroxidase for 10 min. Following that, the sections were covered with 3% goat serum for about 1 hr to block non-specific bindings. The slides were then placed in a humid chamber and incubated for 1 hr with rabbit anti-EGFR polyclonal Abs (Dako, Germany) followed by three washes (2 min each) in buffer. Then, the sections were incubated with biotinylated goat anti-rabbit secondary antibodies (Bio SB, USA) for 30 min, washed three times in buffer, incubated in a Horseradish peroxidase-streptavidin (Envision, Bio SB) for 30 min and washed again four times in buffer. Tissue staining was visualized using the 3, 3′-Diaminobenzidine (DAB) substrate solution (Bio SB, USA) for 10 min and counterstained with hematoxylin. The slides were then dehydrated, mounted and examined by a light microscope (Leica, Germany) to detect the presence of positive immunohistochemistry (IHC) staining in the colonic epithelial cells and macrophages.

A positive EGFR staining was indicated by brown staining of the colonic epithelial cell and cytoplasmic staining of the macrophages in lamina propria and submucosa. Cytoplasmic staining alone (of the colonic epithelial cell), without associated membrane staining, was considered negative. The percentage of positive cells was estimated as 0 (no positive cells), 1 (1–20% positive cells), 2 (21–50% positive cells) and 3 (more than 50% positive cells). The intensity of immunostaining was also estimated as 0 (negative), 1 (weak), 2 (intermediate) and 3 as a strong (19). The intensity level (0-3) was multiplied by the percentage level (0-3) and a final stainig score was assigned as 0 (negative), 1-3 (weak expression), 4-6 (moderate expression), and 7-9 (strong expression) (20).

***Statistical analysis***

The results of the present study are stated as means±SE and the statistical analysis of variation among the experimental groups was performed by the paired T-test and Pearson correlation coefficient test. *P- *values less than 0.05 were considered significant. All statistical exploration was accomplished using SPSS software version 22.

## Results

UUC extent in mice of different groups was assessed at the end of the experiment according to BW changes, the score of DAI, colon length, the score of histological changes and mortalities.

***Body weight changes***

Table 3 shows the changes in BW of the mice on day 1 (starting weight), day 5 (post-DSS exposure weight) and day 16 (post-treatment weight). A significant decrease in BW was apparent on day 5 compared to the starting BW in all groups of mice except those of group 1 (negative control). On day 16, mice of group 2 (positive control “DSS exposure with no treatment”) and group 3 (vehicle control group) still showed a significant weight loss in comparison with their starting BW, whereas mice of group 4 (DSS exposure with 6-TG treatment), group 5 (DSS exposure with 20 mg/kg BW Shogaol treatment), and group 6 (DSS exposure with 40 mg/kg BW Shogaol treatment) regained their normal BW and showed no significant differences compared to their starting BW. This finding revealed that treatment of mice with different concentrations of Shogaol (20 or 40 mg/kg BW) conferred them approximately similar protection to that accomplished by the 6-TG treatment against the weight loss due to the effect of 5% DSS exposure.

***Disease activity index score (DAI score)***

Mice of the negative control group were scored zero, having no symptoms of UC in comparison with the positive control mice, which were scored 12, having prominent blood in their stool, diarrhea and rectal bleeding (Figure 1). On the other hand, the DAI scores of mice in group 3 (control vehicle group “DSS exposure and olive oil treatment”), group 4 (DSS exposure and 6-TG treatment), group 5 (DSS exposure and 20 mg/kg BW Shogaol treatment), and group 6 (DSS exposure and 40 mg/kg BW Shogaol treatment) were 9, 6, 4, and 3 respectively. No mortalities were observed in mice of all groups (Figure 2).

***Average length of the colon***

Table 4 illustrated the average length of the colon in each group of mice at the end of the experiment. In comparison with mice of group 1 (negative control), a significant decrease (*P<*0.05) was shown in colon length in mice of group 2 (positive control) and group 3 (vehicle control group); non-significant decrease was also observed (*P<*0.05) in group 4 (DSS exposure and 6-TG treatment), and group 5 (DSS exposure and 20 mg/kg BW Shogaol treatment), and only minimal decrease was shown in group 6 (DSS exposure and 40 mg/kg BW Shogaol treatment). Representative colon images belonging to mice of the different study groups are shown in Figure 3.

***Histological scoring of ulcerative colitis severity***

The histopathological examination showed inflammation of the colon in mice of the DSS-exposed groups in comparison with the negative control group, which showed normal colon morphology, and the histological scoring (according to extent of epithelial erosions or ulcerations and extent of inflammatory cells infiltration) exhibited different level of colitis severity in the different groups of DSS-exposed mice. In general, the inflammation and the total histological index score were more severe in the distal colon than in the proximal colon in all DSS-exposed mice except those of vehicle and 6-TG treatment groups (Figures 4-6). The highest score (Sum score 6) was recorded for the distal colonic segment in mice of group 2 (control +ve “DSS exposure without treatment”) and the lowest one (Sum score 0) was recorded for the proximal colonic segment in mice of group 6 (DSS exposure and 40 mg/kg BW Shogaol treatment). Histologic score of colitis in mice of the 6-TG treatment group was 2 for both the proximal and distal colonic segment.

***EGFR expression in the colon***

IHC staining of the colonic tissue sections revealed variable scores of EGFR expression in macrophages (within lamina propria and submucosa) and in lining epithelial cells of the mucosal glands in the different groups of mice (Figure 7). Negative expression (Sum score 0) was apparent in mice of the negative control group, strong expression (Sum score 9) in mice of the positive control group, moderate expression (Sum score 6) in mice of the vehicle control group, moderate expression (Sum score 4) in mice of the DSS exposure and 6-TG treatment group, week expression (Sum score 1) in mice of the DSS exposure and 20 mg/kg BW Shogaol treatment group and negative expression (Sum score 0) in mice of the DSS exposure and 40 mg/kg BW Shogaol treatment group.

## Discussion

DSS-induced colitis is one of the most commonly used models that mimics the features of human IBD (21), and is useful to explore novel clinical approaches in colitis treatment (22). 

A significant loss of BW was evident at the end of the experimental duration (on day 16) in mice of the positive control (DSS exposure without treatment) and vehicle control (DSS exposure with olive oil treatment) groups in comparison with the negative control group, whereas mice of the Shogaol treatment groups (especially the 40 mg/kg BW treatment) exhibited BW means approximately comparable with those of the negative control and 6-TG treatment groups. This result indicates that the Shogaol treatment may offer a protective effect against BW loss caused by DSS-induced colitis.

The DAI parameters of colitis (blood in stool, diarrhea and rectal bleeding, clearly evident in mice of the positive control group) were significantly decreased in mice of the 6-TG and Shogaol treatment groups as well as the vehicle control group in comparison with mice of the positive control group. The reduction in DAI score in mice of the latter group (vehicle control group) was probably due to the antioxidant effect of olive oil (23). Interestingly, the DAI scores of the 20 and 40 mg/kg BW Shogaol treatment groups were lower than that of 6-TG treatment group. Similarly, the average colon length was significantly decreased only in mice of the positive control and vehicle control groups compared to that of the negative control group. It was non-significantly decreased in mice of the 6-TG and Shogaol treatment groups. These results demonstrated that the Shogaol treatment has resulted in amelioration of colitis due to its anti-inflammatory effect (24).

Histopathological examination of the colon tissue sections revealed that the DSS exposure succeed in induction of colitis (which appeared to be more severe in the distal than the proximal colonic segment), and the histological scoring of colitis showed that the highest score of severity was recorded for the distal colonic segment in mice of the positive control group and the lowest one was recorded for the proximal colonic segment in mice of the 40 mg/kg BW Shogaol treatment group. Histological score of colitis in mice of the 6-TG treatment group was 2 for both the proximal and distal colonic segment. This finding indicate that the Shogoal treatment might be better than the 6-TG in amelioration of colitis and this is consistent with the finding of Zhang *et al. *(25) who stated that oral delivery of nanoparticles loaded with 6-Shogaol is able to attenuate inflammation of the colon in a murine model of UC.

The results of IHC staining of colonic tissue sections revealed a variable EGFR expression in the different groups of DSS-exposed mice in comparison with the negative control group, which showed negative expression. These results are in agreement with findings of Wright *et al.* (13) and Dubé *et al.* (26) who stated that EGFR signaling plays a central role in the regulation of colon epithelial biology and the response to injury and inflammation. In addition, Lu *et al*. (27) reported that EGFR is activated in colonic macrophages in mice with DSS-induced colitis and in patients with UC. In the groups of DSS-exposed mice, the score of EGFR expression was strong in the positive control group (without treatment), moderate in the vehicle control and 6-TG treatment groups, weak in the 20 mg/kg BW Shogaol treatment group and negative in the 40 mg/kg BW Shogaol treatment group. These findings reveal that the different types of treatments performed in this study have resulted in variable amelioration levels of DSS-induced colitis. The negative EGFR expression in the 40 mg/kg BW Shogaol treatment group compared to the moderate expression in the 6-TG treatment group indicates that the Shogaol is possibly better than the 6-TG in treatment of UC.

## Conclusion

The results of this study revealed that Shogaol, a phenol extract of ginger, has potent curative effects on DSS-induced colitis in the mouse model. The oral 40 mg/kg BW Shogaol treatment boosted the mice health, as indicated by regaining their normal BW and the DAI score, and restored the colonic damage caused by DSS, as indicated by the colon length measurements and scores of histological index and EGFR expression of the colonic tissue sections. These findings may attract the attention regarding the priority of using this cheap plant-derived substance on the chemotherapeutic remedy 6-TG for treatment of the IBD such as UC and Crohn’s disease, because treatment with 6-TG is usually associated with adverse side effects including, hepatotoxicity, nephrotoxicity, and bone marrow suppression leading to anemia, leukopenia and thrombocytopenia (28, 29).
